# Antifungal Activity of *Juglans-regia*-Mediated Silver Nanoparticles (AgNPs) against *Aspergillus-ochraceus*-Induced Toxicity in In Vitro and In Vivo Settings

**DOI:** 10.3390/jfb14040221

**Published:** 2023-04-14

**Authors:** Syeda Itrat Zahra Naqvi, Humera Kausar, Arooj Afzal, Mariam Hashim, Huma Mujahid, Maryam Javed, Christophe Hano, Sumaira Anjum

**Affiliations:** 1 Department of Biotechnology, Kinnaird College for Women, 92-Jail Road, Lahore 54000, Pakistan; 2 Institute of Biochemistry and Biotechnology, University of Veterinary & Animal Sciences, Lahore 54000, Pakistan; 3 Department of Chemical Biology, Eure & Loir Campus, University of Orleans, 28000 Chartres, France

**Keywords:** aflatoxins, antifungal activity, *Aspergillus ochraceus*, hepatotoxicity, silver nanoparticles

## Abstract

Aflatoxins produced by some species of *Aspergillus* are considered secondary toxic fungal by-products in feeds and food. Over the past few decades, many experts have focused on preventing the production of aflatoxins by *Aspergillus ochraceus* and also reducing its toxicity. Applications of various nanomaterials in preventing the production of these toxic aflatoxins have received a lot of attention recently. The purpose of this study was to ascertain the protective impact of *Juglans-regia*-mediated silver nanoparticles (AgNPs) against *Aspergillus-ochraceus*-induced toxicity by exhibiting strong antifungal activity in in vitro (wheat seeds) and in vivo (Albino rats) settings. For the synthesis of AgNPs, the leaf extract of *J. regia* enriched with high phenolic (72.68 ± 2.13 mg GAE/g DW) and flavonoid (18.89 ± 0.31 mg QE/g DW) contents was used. Synthesized AgNPs were characterized by various techniques, including TEM, EDX, FT-IR, and XRD, which revealed that the particles were spherical in shape with no agglomeration and fine particle size in the range of 16–20 nm. In vitro antifungal activity of AgNPs was tested on wheat grains by inhibiting the production of toxic aflatoxins by *A. ochraceus*. According to the results obtained from High-Performance Liquid Chromatography (HPLC) and Thin-Layer Chromatography (TLC) analyses, there was a correlation between the concentration of AgNPs and a decrease in the production of aflatoxin G1, B1, and G2. For in vivo antifungal activity, Albino rats were administrated with different doses of AgNPs in five groups. The results indicated that the feed concentration of 50 µg/kg feed of AgNPs was more effective in improving the disturbed levels of different functional parameters of the liver (alanine transaminase (ALT): 54.0 ± 3.79 U/L and aspartate transaminase (AST): 206 ± 8.69 U/L) and kidney (creatinine 0.49 ± 0.020 U/L and BUN 35.7 ± 1.45 U/L), as well as the lipid profile (LDL 22.3 ± 1.45 U/L and HDL 26.3 ± 2.33 U/L). Furthermore, the histopathological analysis of various organs also revealed that the production of aflatoxins was successfully inhibited by AgNPs. It was concluded that the harmful effects of aflatoxins produced by *A. ochraceus* can be successfully neutralized by using *J. regia*-mediated AgNPs.

## 1. Introduction

Mycotoxins are secondary metabolites that are produced by toxigenic strains of fungus and can contaminate a number of food commodities. The most frequently found mycotoxins in major crops are produced by genera *Aspergillus*, *Penicillium*, and *Fusarium*. The occurrence of mycotoxins in cereals and other food commodities is of greatest concern due to their significant impact on animal and human health. Among these mycotoxins, ochratoxin A, aflatoxin B1, and fumonisin B1 are more toxic to mammals [1]. Aflatoxins are mycotoxins produced by *Aspergillus* spp., particularly by *Aspergillus flavus* and *Aspergillus paraciticus* [2]. Chemically, aflatoxins are difuranocoumarin derivatives and are known for inducing mutagenicity, hepatotoxicity, teratogenicity, cytotoxicity, immunosuppression, and estrogenic effects in mammals [3,4,5]. Naturally occurring major aflatoxins are aflatoxin B1, aflatoxin G1, aflatoxin B2, and aflatoxin G2 [6]. Aflatoxins contaminate a variety of food commodities, including wheat, maize, rice, fig, peanut, peanut butter, cocoa, spices, millet, and sesame seeds [7]. The consumption of food contaminated with aflatoxins may lead to serious health issues, including severe damage to the liver, heart, and kidney, which can lead to hemorrhage or even death [8].

In light of prior findings, it is vital to create a variety of physical, chemical, and biological approaches for the detoxification of mycotoxins. The employment of antimicrobial nanoparticles to overcome this problem is one of the most promising options [9]. Silver, in this regard, is considered as a potent antimicrobial agent, available naturally and shows less toxicity in mammalian tissues [10]. Silver is known for its ability to attack a number of metabolic events in microorganisms including the modification of the structure and functions of the cell membrane [11,12,13]. It also hinders the protein expression associated with the production of ATP [14]. Different studies have been conducted on the antibacterial activity exhibited by silver [15,16,17] leading to the wide application of silver nanoparticles (AgNPs) in the purification of water, washing machines, shampoo, toothpaste, cooking utensils, and toys [18]. AgNPs are increasingly being used as an antimicrobial agent due to their strong antibacterial and antifungal properties [19].

Nanoparticles (NPs) can be synthesized in a variety of ways. Because of its environmentally friendly and cost-effective nature, green synthesis of nanoparticles has gained a lot of interest in recent years [20,21,22]. NPs synthesized using standard procedures are limited in therapeutic applications because of their adverse effects. Thus, green-synthesized NPs have an edge over other NPs in terms of lifespan, physical qualities, and chemical properties [23]. In this study, the leaf extract of the walnut tree (*Juglans regia* L.) has been used to synthesize AgNPs, as it possesses a significant amount of phenolic compounds, which have powerful antioxidant and antimicrobial properties [24,25,26]. *J. regia* is widely distributed throughout Europe, the Middle East, and North Africa and is now cultivated in many other regions of the world. In addition to its culinary uses, *J. regia* has a long history of medicinal use as it includes a variety of nutrients, including healthy fats, protein, and antioxidants, which are believed to have a range of health benefits [27,28,29]. Studies have shown that *J. regia* may help reduce inflammation, improve heart health, and protect against certain types of cancer. It is also used in traditional medicine to treat conditions, such as asthma, eczema, and constipation [30,31]. Different components of the walnut plant have been investigated for the treatment of multiple diseases and microbial infections [24]. Thus, the bioactive substances included in *J. regia* leaf extract can function as a metal ion reducer, as well as a capping and stabilizing agent for the fabrication of various kinds of NPs.

The current study highlights the use of *J. regia*-mediated AgNPs to prevent the production of toxic aflatoxins by *Aspergillus ochraceus* in both in vitro and in vivo settings. One of the key novelties of this study is the demonstration that biologically synthesized AgNPs are strong antifungal agents against *A. ochraceus* and can protect various organs against toxicity caused by aflatoxins. The study also provides new insights into the potential of nanomaterials for preventing the production of toxic fungal by-products in feeds and food. Thus, AgNPs can be potentially employed in preventing the harmful effects of aflatoxins on human health and animal production. Further research incorporating in silico/computational simulation studies could greatly enhance our understanding of the mechanisms underlying the interactions between mycotoxins, nanoparticles, and biological systems. The urgency of conducting such studies is high due to several advantages that computational simulations offer compared to traditional in vivo and in vitro experiments, including lower costs and faster results [32].

## 2. Materials and Methods

### 2.1. Preparation of J. regia Leaf Extract

Fresh leaves of *J. regia* were obtained from the nursery of Kinnaird College for Women University in Lahore. To remove any dust or contaminants, 10 g of fresh leaves was weighed and thoroughly washed with distilled water. The leaves were then chopped and boiled in 400 mL of distilled water under sterile conditions. The resulting mixture was then condensed to a final volume of 100 mL by boiling. The mixture was filtered through Whatman filter paper, and the resulting filtrate was collected and stored in an airtight jar at a temperature of 4 °C for further use.

### 2.2. Synthesis of Silver Nanoparticles Using Leaf Extract of J. regia

AgNPs were synthesized according to the method described by Ravichandran et al. [33]. Initially, the leaf extract of *J. regia* was mixed with 0.5 mM AgNO_3_ solution (SNS) in different ratios (1:2 *v*/*v*, 1:4 *v*/*v*, 1:6 *v*/*v*, and 1:8 *v*/*v*). After subjecting the reaction mixtures with ratios of 1:6 *v*/*v* and 1:8 *v*/*v* to shaking for 10 min, a noticeable change in color from yellowish to reddish brown was observed. However, no significant color change was observed in reaction mixtures with ratios of 1:2 *v*/*v* and 1:1 *v*/*v* even after an hour. By measuring the Surface Plasmon Resonance (SPR) with UV visible spectroscopy, AgNPs production was observed. The 1:6 *v*/*v* reaction mixture demonstrated rapid AgNPs production (within 10 min) and higher stabilization when compared to the 1:8 *v*/*v* and 1:2 ratios *v*/*v*. On the other hand, there was no significant color change or SPR peak was observed in the 1:1 *v*/*v* ratio. Therefore, the ratio 1:6 *v*/*v* was selected for the further formation of AgNPs in bulk. The reaction mixture (1:6 *v*/*v*) was then centrifuged for 15 min at 13,000 rpm. The pellets were then washed, centrifuged, and dried at room temperature, resulting in powdered AgNPs formation.

### 2.3. Phytochemical Analysis of J. regia Leaf Extract

#### 2.3.1. Estimation of Total Phenolic and Flavonoid Content

The total phenolic content (TPC) of *J. regia* leaf extract (LE) was assessed using Folin–Ciocalteu’s method as described by Velioglu et al. [34]. A UV-Vis spectrophotometer (Shimadzu, Kyoto, Japan, UV-3600) was employed to measure the absorbance at 725 nm. Using Gallic acid as a standard, TPC was expressed as mg Gallic acid equivalent (GAE)/g dry weight of plant extract. All experiments were performed in triplicates.

The total flavonoid content (TFC) was determined by using the aluminum chloride colorimetric method as demonstrated by Chang et al. [35]. Quercetin was used as a standard and for plotting the calibration curve at the concentration of 0–200 µg/mL. TFC was expressed as Quercetin equivalent (QE, mg Quercetin/g dry weight of plant extract). All determinations were performed in triplicate.

#### 2.3.2. 2,2-Diphenyl-1-picrylhydrazyl (DPPH)

The free radical scavenging activity (FRSA) was carried out using the methodology suggested by Abbasi et al. [36]. The tested samples (20 µL of the plant extract stock solution) were introduced into a microtiter plate, followed by the addition of DPPH reagent (180 µL). The mixture was maintained at room temperature and in the dark for 60 min. As a negative control, concentrations of ascorbic acid (05, 10, 20, and 40 g/mL), DMSO (20 µL), and DPPH (180 µL) were used. The absorbance of the resulting solution was measured at 517 nm using a microplate reader.

### 2.4. Characterization of J. regia-Mediated Silver Nanoparticles

The bio-reduction of pure Ag^+^ ions into AgNPs was monitored using a UV-Visible spectrophotometer. The reaction mixtures were analyzed every 30 min for 24 h, and UV-Vis spectra were taken between 350 and 700 nm to record the synthesis of AgNPs. The Fourier-transform infrared (FTIR) spectra of *J. regia* and AgNPs were performed as described by Tungmunnithum et al. [37]. The possible functional groups involved in the capping and reduction of AgNPs were determined using the reflectivity mode of a Burcker V70 interferometer within the 400 and 4500 cm^−1^ wavenumber range. The values obtained from FTIR were the mean of triplicates.

The morphology of *J. regia*-mediated AgNPs was determined using Transmission Electron Microscopy (TEM) (JEM-1011; JEOL, Kyoto, Japan). TEM pictures of AgNPs at various magnifications were acquired using a dilute suspension of samples dropped on a carbon-coated copper grid and dried for 5 min under a mercury lamp. The elemental analysis of AgNPs was carried out using an Energy-Dispersive X-ray Spectroscopy (EDX) (JEOL JEM-ARM200 F 200 kV) detector mounted to a TEM. The crystalline nature of synthesized AgNPs was determined using X-Ray Diffraction (XRD) (Shimadzu-Model, Kyoto, Japan, XRD 6000). AgNPs were coated on an XRD grid, and measurements were taken using an X-ray diffractometer operating at 40 kV. The size of AgNPs was calculated using the Debye–Scherrer equation [38]. Dynamic Light Scattering (DLS) was utilized with the aid of Zetasizer (NanoZSP, Malvern, UK) to measure the size distribution of AgNPs as described by Sohail et al. [39].

### 2.5. Collection and Growth of Fungus

Identified culture of toxigenic *Aspergillus ochraceus* (CECT 2948) was procured from the Institute of Microbiology, University of Veterinary and Animal Sciences (UVAS), Lahore, Pakistan. The culture was revived by culturing and subculturing on Sabouraud’s Dextrose Agar plates (SDA) and slants. Agar plates and slants were incubated at 28 °C for 10 to 14 days. Pure colonies of fungus *A. ochraceous* were obtained and stored in a refrigerator for further experimental analysis.

### 2.6. In Vitro Antifungal Activity of Silver Nanoparticles

#### 2.6.1. Collection and Sampling of Wheat for In Vitro Antifungal Activity

The inoculation of *Aspergillus ochraceus* (AO) was conducted according to the procedure demonstrated by Trenk et al. [40]. Wheat grains were purchased from the local market. Antifungal and antitoxin activity of AgNPs was analyzed by making eight groups in Erlenmeyer flasks. Wheat grains (50 g) were soaked in an equal volume of distilled water (50 mL) in each flask. After 2 h, flasks were autoclaved at 121 °C and 15 psi for 20 min. Flasks were inoculated with fungal culture (1 × 10^6^ spores/mL) of *A. ochraceus* with varying concentrations of NPs, i.e., group A (50 g wheat + AO), group B (50 g wheat + AO + 10 µg AgNPs), group C (50 g wheat + AO + 20 µg AgNPs), group D (50 g wheat + AO + 30 µg AgNPs), group E (50 g wheat + AO + 40 µg AgNPs), group F (50 g wheat + AO + 50 µg AgNPs), group G (50 g wheat + AO + 60 µg AgNPs), and group H (50 g wheat + AO + 70 µg of AgNPs) of AgNPs in a sterilized environment to avoid contamination.

#### 2.6.2. Extraction and Estimation of Toxins

**Figure 1:**
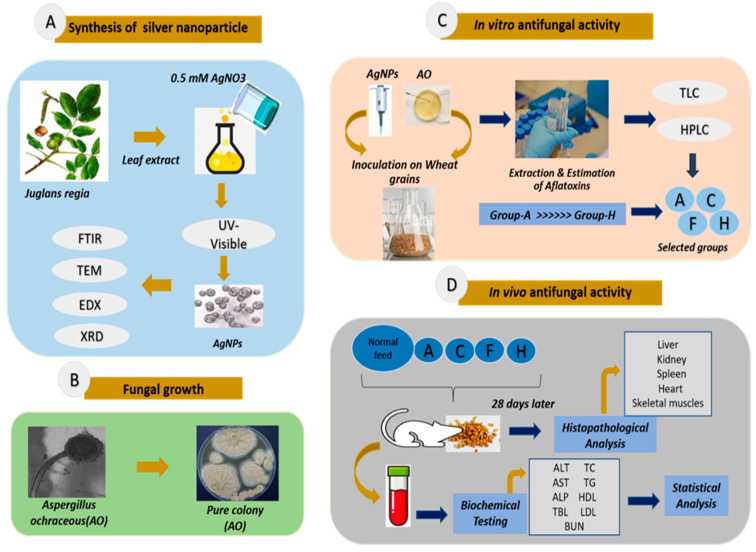
Workflow for antifungal activity of AgNPs against A. ochraceus-induced toxicity in in vitro and in vivo settings. (**A**) Synthesis of AgNPs; AgNPs were synthesized from leaf extract of *J. regia*. (**B**) Fungal growth; a pure colony of toxigenic fungus A. ochraceous was obtained from culturing and subculturing. (**C**) In vitro antifungal activity; the experiment consisted of eight groups, where group A served as the control group (50 g wheat + AO) and group B (50 g wheat + AO + 10 µg AgNPs), group C (50 g wheat + AO + 20 µg AgNPs), group D (50 g wheat + AO + 30 µg AgNPs), group E (50 g wheat + AO + 40 µg AgNPs), group F (50 g wheat + AO + 50 µg AgNPs), group G (50 g wheat + AO + 60 µg AgNPs), and group H (50 g wheat + AO + 70 µg AgNPs). (**D**) In vivo antifungal activity; five groups of albino rats were distributed as group A (control with normal feed), group B (fed with wheat from group A), group C (fed with wheat from group C), group D (fed with wheat from group F), and group E (fed with wheat from group H). After a 28-day trial, biochemical testing of blood was performed, and one-way analysis of variance (ANOVA) followed by Bonferroni’s test was used to compare the weight gain, food consumption, and results of hematological and biochemical parameters. *p*-values < 0.05 were considered as significant, and histopathological examination of the liver, kidney, spleen, skeletal muscle, and heart was carried out and observed under light microscopy. Note: AgNO_3_, silver nitrate; AgNPs, silver nanoparticles; UV-Visible, UV-Vis spectrophotometer; FTIR, Fourier-transform infrared spectroscopy; TEM, transmission electron microscopy; EDX, energy-dispersive X-ray spectroscopy; XRD, X-ray diffraction; AO, Aspergillus ochraceous; TLC, thin-layer chromatography; HPLC, high-performance liquid chromatography; ALT, alanine aminotransferase; AST, aspartate aminotransferase; ALP, alkaline phosphatase; TBL, total bilirubin; BUN, blood urea nitrogen; creatinine; TC, total cholesterol; TG, triglycerides; HDL, high-density lipoprotein; LDL, low-density lipoprotein.

Toxins produced by AO were extracted by solvent extraction in acetonitrile–water as described by Bayman et al. [41]. Briefly, 5 g of the fermented wheat grains was mixed with 20 mL of acetonitrile–water (70:30) and was placed on a shaking incubator for 2 h. The mixture was filtered using Whatman’s filter paper (0.45 µm). The filtrate was subjected to TLC for identification, and quantification was executed by HPLC (Agilent 1100 system) using standard AF (Sigma Aldrich, Saint Louis, MO, USA, CAS no: A6636) and C18 Column, where the mobile phase was acetonitrile:methanol:water (20:20:60; *v*/*v*/*v*). The entire methodology is summarized in Figure 1.

### 2.7. In Vivo Antifungal Activity of Silver Nanoparticles

#### 2.7.1. Study Design

Eight-week-old 25 albino rats were divided into five groups (A–E); each group containing 5 rats. The three different doses of AgNPs were selected for comparative evaluation on the basis of the results of in vitro antifungal activity against *A. ochraceous* in grown wheat as confirmed by HPLC. Group A was kept as a control group, group B (only toxin in feed), group C (toxin + 20 µg AgNPs in feed), group D (toxin + 50 µg AgNPs in feed), and group E (toxin + 70 µg AgNPs in feed). The trial was conducted at UVAS, Lahore, Punjab, Pakistan, for a total period of 28 days. The Ethical Review Committee, UVAS, Lahore, granted authorization for all experimental work. The concentration of fungal spores and AgNPs was selected under the OECD test guideline 407 (OECD, Paris, France, 1995). Increase in weight, feed consumption, and FCR were measured on a weekly basis.

#### 2.7.2. Biochemical Testing

Rats were dissected and blood (2 mL) was obtained at the end of the trial by an expert veterinarian in red top vacutainers. The serum was separated from the blood and kept at a temperature of −20 °C. The activity of alanine transaminase (ALT), aspartate transaminase (AST), alkaline phosphatase (ALP), total bilirubin (TBL), blood urea nitrogen (BUN), creatinine, total cholesterol (TC), triglycerides (TG), high-density lipoprotein (HDL), and low-density lipoprotein (LDL) was analyzed using a chemistry analyzer at Quality Operation Laboratory, UVAS, Lahore.

#### 2.7.3. Histopathological Analysis

Histopathological examination of the liver, kidney, spleen, skeletal muscle, and heart was carried out as explained by Bancroft and Cook [42]. Briefly, organs were removed and immediately stored in 10% formalin. The sections of organs were fixed with paraffin, stained using hematoxylin and eosin, and observed under light microscopy.

### 2.8. Statistical Analysis

The one-way analysis of variance (ANOVA) followed by Bonferroni’s test was used to compare the weight gain, food consumption, and results of hematological and biochemical parameters. *p*-values < 0.05 were considered as significant.

## 3. Results and Discussion

### 3.1. Determination of Phytochemical Content

*J. regia*, a medicinal plant with great therapeutic potential, has been widely used to treat diabetes, cancer, rheumatic pain, infectious diseases, and fever [43,44,45]. It is also known to possess a range of bioactive compounds, such as phenolics, flavonoids, terpenoids, tannins, and saponins, that may be involved in the reduction of Ag^+^ to Ag^0^ during the synthesis of AgNPs [46,47]. Phytochemical analysis of the aqueous extract of *J. regia* showed that it possesses a significant amount of TPC (72.68 ± 2.13 mg GAE/g DW) and TFC (18.89 ± 0.31 mg QE/g DW). Nabavi et al. [48] reported the TPC of *J. regia* to be 71.7 ± 3.2 mg GAE/g, which shows similarity with our results. Likewise, Oliveira et al. [49] also reported the TPC of *J. regia* green husk to be 74.08 mg/g of GAE, and our results are in accordance with it. In the case of flavonoids, Yang et al. [50] reported the TFC of *J. regia* ethanolic extract as 18.97 ± 0.21 mg/g, and our results are in close proximity. Similarly, these findings are also in accordance with the study published by Shah et al. [51] who reported the TFC of walnut leaf extract in the range of 5.52 to 28.48 mg QE/g. It is thus speculated that phytochemicals such as flavonoids, polyphenols, and alkaloids might be involved in the reduction, capping, and stabilization of AgNPs [52,53], as phenolics, flavonoids, reducing sugars, and proteins found in almost all medicinal plants have been demonstrated to function as bio-reductants of metallic ions in aqueous medium [54,55].

A stable free radical DPPH is used frequently in chemical analysis to measure free radical scavenging activity [56]. The results display that the leaf extract of *J. regia* exhibits excellent antioxidant activity (87.52 ± 1.97 %). This value is closer to 83.16% as per the results demonstrated by Oliveira et al. [49]. In addition, Slatnar et al. [57] showed the scavenging activity of kernels to be in the range of 60.0% to 96.4% which shows a close accordance with our results. Overall, the findings point to the existence of enhanced flavonoid and phenolic compounds, which serve to boost antioxidant activity and, as a consequence, produce NPs with high yields.

### 3.2. Synthesis of J. regia-Mediated AgNPs

**Figure 2:**
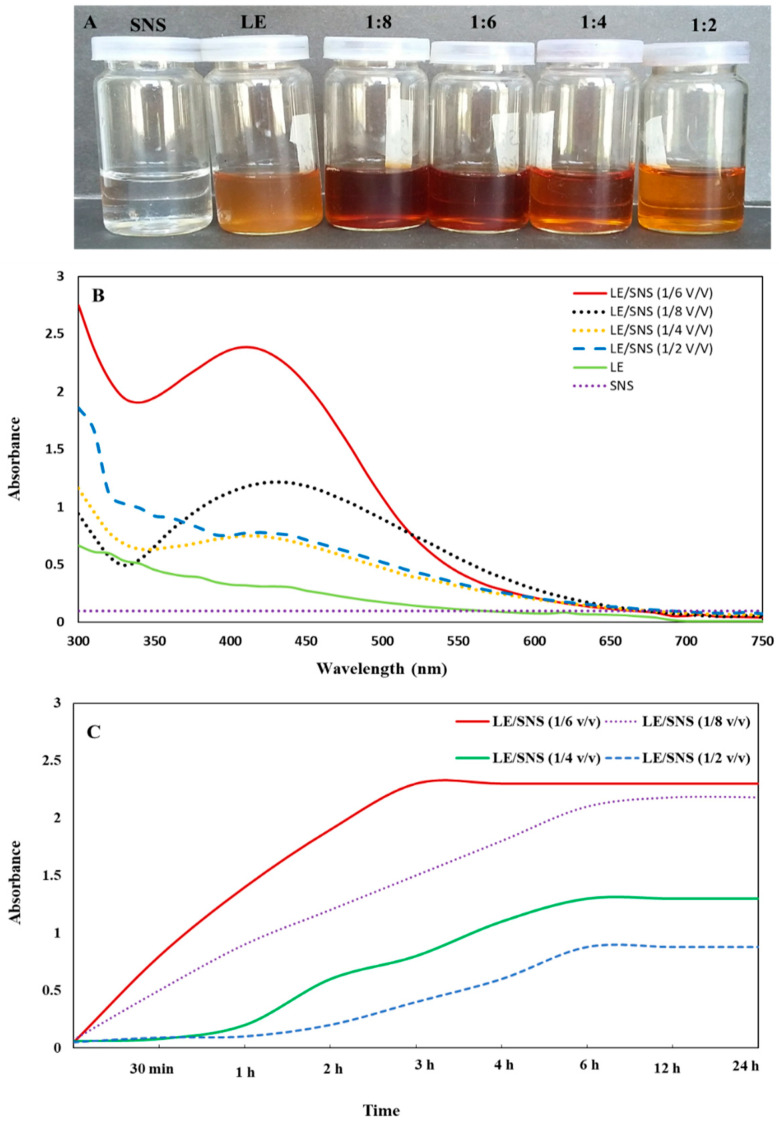
(**A**) Reaction mixtures of LE/SNS in different ratios (*v*/*v*). (**B**) UV-Vis spectra of *J. regia*-mediated AgNPs. (**C**) Absorbance profiles of *J. regia*-mediated AgNPs at different time intervals.

The initial observation of a visible color shift from yellowish brown to dark brown in the reaction mixture of aqueous LE and SNS can be regarded as a reliable indicator to monitor the progression of AgNPs formation [58,59]. To investigate the formation of AgNPs, 100 µL of an aqueous extract of *J. regia* was combined with 1 mM of sodium nitrate solution (SNS) at different *v*/*v* ratios (1:2, 1:4, 1:6, 1:8; aqueous extract/SNS), and the resulting mixture was incubated at room temperature for 24 h. Visual inspection revealed a color change to brown in the reaction mixtures, while no color change was observed in either the aqueous extract or SNS solutions alone. The results of the experiment indicate that the reaction mixtures containing 1:6 and 1:8 *v*/*v* ratios (aqueous extract/SNS) demonstrated the highest degree of color intensity when compared to the other reaction mixtures tested. This suggests that the reduction of Ag^+^ ions to AgNPs was more pronounced in these mixtures, as illustrated in Figure 2A. Higher concentrations of SNS solution accelerated AgNPs production, which is consistent with a prior work using *Echinacea purpurea* extract as a reducing agent [60]. The primary indication of the formation of AgNPs is a discernible alteration in color, which arises from the pronounced absorption of visible light, leading to the excitation of surface plasmon resonance in AgNPs [59,61].

The exact mechanism involved in the formation of plant-mediated AgNPs is still unknown. Phytochemical analysis of *J. regia*-mediated leaf extract revealed a high content of TPC (72.68 ± 2.13 mg GAE/g DW) and TFC (18.89 ± 0.31 mg QE/g DW). Similar to our results, high TPC and TFC were found to be present in the leaf and bark extract of *J. regia* [62,63]. The FTIR examination revealed the presence of compounds, such as alkyl, amide, phenolic, and flavonoid, that were further involved in the reduction and capping of the synthesized AgNPs. Hence, we propose a redox process as the mechanism for reducing silver ions utilizing phenolics and flavonoids. Silver ions in plant extract first combine with active components of phenolics and flavonoids to produce an intermediate silver complex, which is then oxidized into keto form with the release of free electrons and Ag^+^ ions. In the presence of free electrons generated during the reduction process, these Ag^+^ ions are then converted to zero-valent silver (Ag^0^). Nitrate ions and unreacted secondary metabolites from plants are by-products of the reduction reaction that are eliminated during the washing of AgNPs [54,64]. This method is in line with earlier reports on the reduction and stabilization of the plant-mediated production of AgNPs [54,65,66,67].

### 3.3. Characterization of J. regia-Mediated Silver Nanoparticles

#### 3.3.1. UV-Visible Spectroscopy

UV-Visible (UV-Vis) spectroscopy is a vital analytical technique used for evaluating the formation and stability of AgNPs in aqueous solutions. Literature supports that AgNPs display a distinctive surface plasmon resonance (SPR) peak within the wavelength range of 400–460 nm, which can be employed for characterization purposes [68,69]. Figure 2B depicts the SPR in the 440–450 nm region, confirming the bio-reduction of Ag^+^ to AgNPs. Different SPR peaks of reaction mixtures (LE mixed with SNS) were recorded at different ratios; however, the peak at 1:6 *v*/*v* ratio shows the formation of stable NPs with higher absorbance. The rise in absorbance intensity is attributable to an increase in the yield of AgNPs as silver ions are reduced [55,70]. It is widely known that the absorbance intensity of AgNPs is mostly determined by their size and shape, and that absorbance peaks generally drop as the size of the NPs increases [69]. Hawar et al. [71] conducted a similar study in which AgNPs showed an SPR at 435 nm, which is a characteristic peak of AgNPs. Likewise, green-synthesized AgNPs from an aqueous extract of *Moringa oleifera* showed a peak at 440 nm, and our results are in accordance with it [72]. In another study, AgNPs synthesized using leaf extract of *Caesalpinia bonducella* represented an SPR at 427 nm, indicating the formation of oval and spherical-shaped NPs [73]. The synthesis of AgNPs was demonstrated using the leaf extract obtained from *Rhaphidophora pertusa*. The successful synthesis of AgNPs was confirmed by observing the SPR peak at 424 nm, which is indicative of the presence of AgNPs and is in close proximity to our results [74].

Absorbance intensities and wavelength peaks of reaction mixtures were also recorded at different time intervals (30 min−24 h) to calculate the total duration period required to reduce silver ions and the time to acquire stability. Figure 2C depicts the increase in absorbance intensities in 1:2 *v*/*v*, 1:4 *v*/*v*, and 1:8 *v*/*v* reaction mixture of LE/SNS up till 6 h; however, no significant change was observed after 6 h, which indicates the completion of the reduction process in 6 h in 1:8 *v*/*v* and 1:2 *v*/*v* ratios. In the case of a 1:6 *v*/*v* ratio mixture, the reduction was completed within 3 h, which indicates a higher yield in a short time. This might be due to the higher stability of AgNPs in 1:6 *v*/*v* than in the 1:8 *v*/*v* reaction mixture. Similar results were reported by Devanesan et al. [75] and Jabir et al. [76] using extract of *Abelmoschus esculentus* and *Annona muricata* for the synthesis of AgNPs. After observing that the reaction mixture with a 1:6 *v*/*v* ratio displayed a narrow peak at 440 nm, indicating a higher yield and stability within a short timeframe, and no shift in wavelength occurred after being stored at room temperature for two months, we decided to choose this ratio for the bulk synthesis and to study the biological activities of the resulting AgNPs.

#### 3.3.2. Transmission Electron Microscopy (TEM) and Energy-Dispersive X-ray (EDX) Spectroscopy

**Figure 3:**
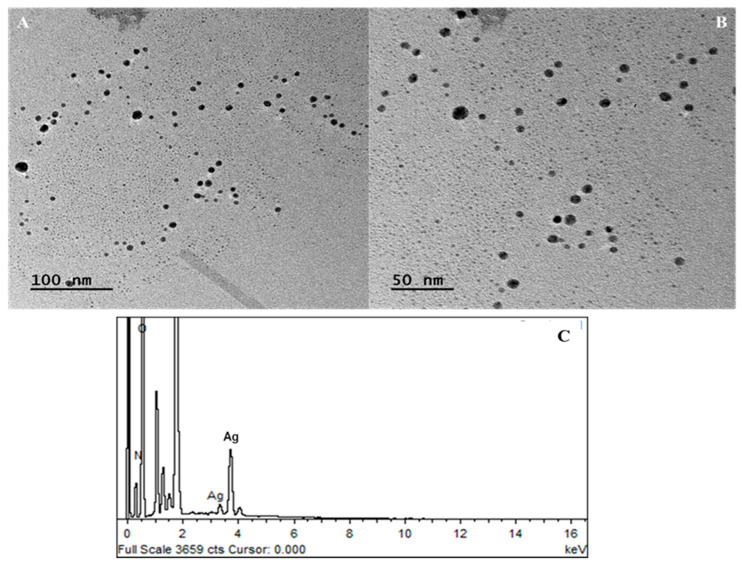
(**A**) TEM images of *J. regia*-mediated AgNPs at 100 nm. (**B**) TEM images of *J. regia*-mediated AgNPs at 50 nm. (**C**) EDX spectra of *J. regia*-mediated AgNPs.

The TEM technique was employed to determine the morphology and structure of green-synthesized AgNPs. TEM images at the magnification of 50,000× and 100,000× revealed the spherical morphology of AgNPs with a polydispersity nature and no agglomeration as shown in Figure 3A and B. Moreover, size calculations using Sigma Scan Pro software (Systat Software Inc., San Jose, CA, USA) in conjunction with TEM revealed that AgNPs were in the range of 16 ± 2 nm, and our results are in accordance with the study conducted by Suriyakala et al. [77]. Likewise, a study conducted by Pei et al. [78] reported the formation of spherical-shaped AgNPs using an aqueous extract of *Coptis chinensis*. Moreover, Khan et al. [79] also reported the formation of spherical AgNPs using leaf extract of *Hibiscus sabdariffa*. EDX analysis was carried out to further confirm the presence of metallic silver in the AgNPs. The EDX spectra as shown in Figure 3C revealed prominent metallic silver peaks in the 3.0–4.0 keV region. Metallic silver nanocrystals often have significant absorption spectra in the 2.5–4.0 keV range, and similar results have previously been reported by Kapoor et al. [80] depicting characteristic peak of silver at 3.0 keV. Sengottaiyan et al. [81] also demonstrated a peak of silver at 3.0 keV synthesized from *Solanum indicum* leaf extract. The spectrum obtained from the analysis of AgNPs synthesized from *Ribes Rubrum* displayed a distinct absorption peak at 3 keV, which shows similarity with our results [82].

#### 3.3.3. Fourier-Transform Infrared Spectroscopy (FT-IR)

**Figure 4:**
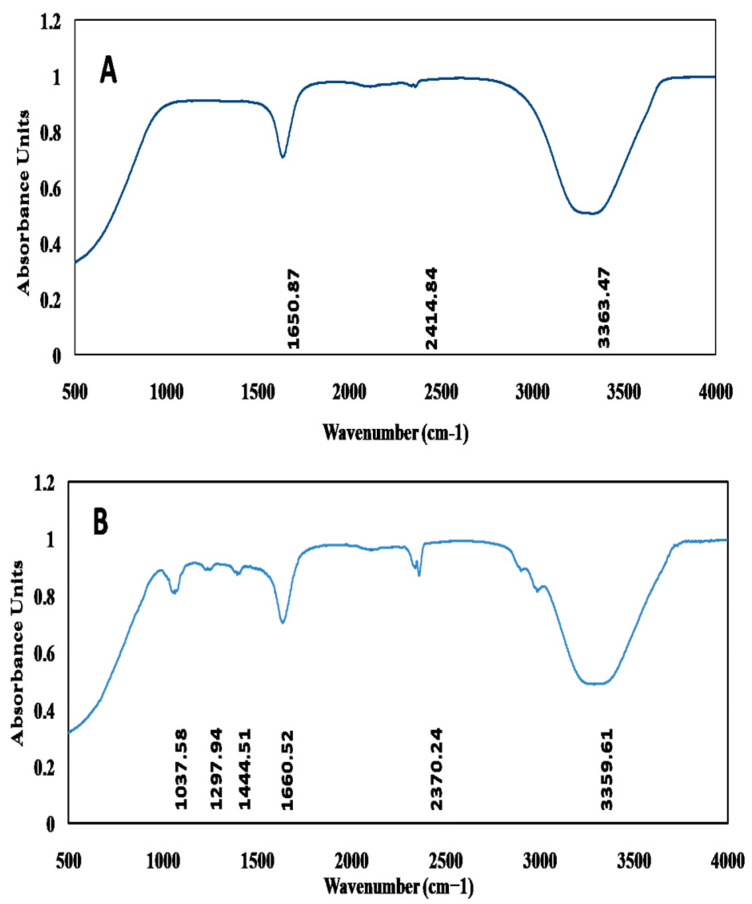
FTIR spectrum of (**A**) *J. regia*-mediated AgNPs and (**B**) *J. regia* LE.

FTIR analysis of both the *J. regia* LE and *J. regia*-mediated AgNPs was carried out to investigate the possible phytochemicals involved in the reduction, capping, and stabilization of AgNPs. The findings revealed the intense absorption peaks at 1650.87, 2414.84, and 3363.47 cm^−1^ for *J. regia*-mediated AgNPs (Figure 4A), whereas for *J. regia* LE absorption peaks were recorded at 1037.58, 1297.94, 1444.51, 1660.52, 2370.24, and 3359.61 cm^−1^, respectively (Figure 4B). The observed peaks were compared with standard values to confirm the presence of functional groups. The absorption peak at 1037.58 cm^−1^ represents the stretching vibrations of the C-O bond [83]. The absorption band at 1297.94 cm^−1^ corresponds to the C-N stretching of aliphatic amines [61], whereas the peak at 1444.51 cm^−1^ depicts the stretching of C-H in alkenes. The intense peaks in the range of 1660.52–1650.87 cm^−1^ correspond to the stretching of nitrile groups [84], and absorption peaks at 2370.24–2414.84 cm^−1^ represent the stretching of N-H groups [85]. Lastly, the peak in the range of 3359.61–3363.47 cm^−1^ was assigned to the stretching of –OH in phenolic and alcoholic compounds [54]. Both the LE and AgNPs shared common peaks in the range of 1660.52–3363.47 cm^−1^, which clearly indicates the presence of the same phytochemicals as present in LE involved in the capping, reduction, and stabilization of AgNPs. The present data strongly suggest the presence of alkyl, amide, phenolic, and flavonoid compounds present in LE extract which further reduced and capped the synthesized AgNPs.

#### 3.3.4. X-ray Diffraction Analysis (XRD)

**Figure 5:**
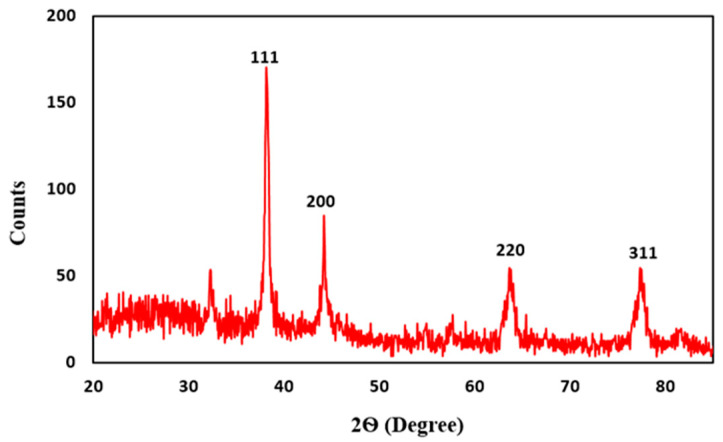
XRD pattern of *J. regia*-mediated AgNPs.

XRD analysis was employed to determine the crystalline nature of *J. regia*-mediated AgNPs. Figure 5 shows the presence of four diffraction peaks having 2θ angle at 38.31°, 44.37°, 65.41°, and 77.29°, which can be attributed to 111, 200, 220, and 311 crystallographic planes of the face-centered cubic (FCC) crystalline AgNPs [61]. FCC structure of AgNPs at 111, 200, 220, and 311 planes was observed by Abbasi et al. [53], and our results show similarity with it. Likewise, Nahar et al. [86] showed three intense peaks at 38.44°, 44.3°, and 64.82° with planes of 111, 200, and 220, thereby representing the FCC structure of AgNPs and our results are in accordance with it. Fouda et al. [87] also reported the FCC structure of *Aspergillus-flavus*-mediated AgNPs which is in accordance with our findings. The average size of the synthesized AgNPs calculated using the Debye–Scherrer equation appeared to be 20.67 nm and is in accordance with the size calculated by TEM and the previous reports available in the literature [88,89,90].

#### 3.3.5. Dynamic Light Scattering (DLS) and Zeta Potential Analysis

**Figure 6:**
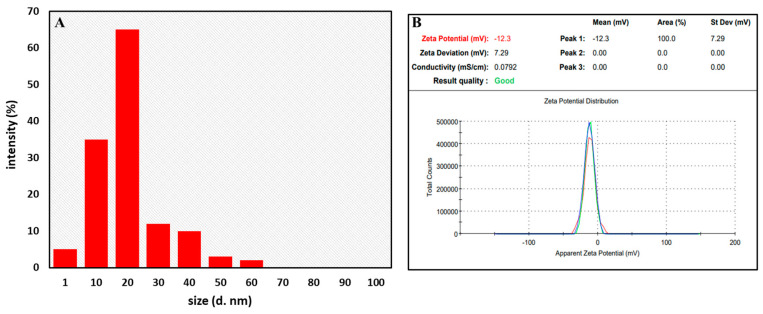
(**A**) DLS and (**B**) zeta potential analysis of AgNPs.

DLS offers a measurement of the size distribution of NPs from the scattered light in a solution [91]. Figure 6A represents the size distribution of AgNPs measured by DLS which appeared to be 18 nm. These data are in accordance with the size reported from XRD and TEM. Parvataneni et al. [92] showed the average size of AgNPs synthesized from *Scoparia dulcis* leaf extract to be 8.2 nm as measured from the DLS technique. The average size of the synthesized AgNPs from Oak Fruit Hall from DLS also appeared to be 40 nm [93]. In addition, Manjunath and Chandrashekhar [94] also reported the average size of AgNPs to be in the range of 30–60 nm as measured by the DLS technique. Thus, the size distribution of AgNPs as shown by DLS is consistent with the size estimated through TEM and XRD.

The zeta potential of biosynthesized AgNPs was discovered to be −12.3 mV, with a strong peak (Figure 6B). The surface of the NPs may be negatively charged and dispersed in the medium [95,96]. The fact that the value is negative demonstrates that the AgNPs were highly stable. This outcome is in line with AgNPs synthesized from leaf extracts of other plants [97,98].

### 3.4. In Vitro Antifungal Activity of AgNPs

*In-vitro* antifungal activity was determined through TLC and HPLC. Under UV illumination (366 nm), TLC plates showed fluorescence of aflatoxins in the standard group, whereas groups C−H did not fluoresce when observed under UV, which confirmed the antifungal activity of AgNPs. On the basis of the results of TLC, HPLC of the selected groups (group C−H) was further performed to validate the antifungal activity of AgNPs. The results showed the presence of aflatoxin G1 (363 ± 3.06 ng/mL), B1 (9.74 ± 0.541 ng/mL), and G2 (54.4 ± 3.95 ng/mL) in the control samples with fewer amounts of G1 and absence of B1 and G2 in groups F–H (Table 1). AgNPs in group B (10 µg), group C (20 µg), and group D (30 µg) did not show any reduction in the production of G1, B1, and G2. However, group E (40 µg), group F (50 µg), group G (60 µg), and group H (70 µg) showed an increase in the production of aflatoxin G1 with the absence of B1 and G2, thus attributing to the stronger antifungal potential of AgNPs upon increasing concentrations. A similar study was conducted by Ibrahim et al. [99] which showed the suppression of mycelium growth employing AgNPs at concentrations of 5 (45.56%), 10 (62.22%), 15 (72.78%), and 20 (80.56%) µg/mL. Likewise, Bocate et al. [100] also showed the antifungal activity of AgNPs against toxigenic species of *Aspergillus ochraceus*, *Aspergillus melleus*, and *Aspergillus flavus*, thus suggesting the good potential of AgNPs to act as an antifungal agent.

### 3.5. In Vivo Antifungal Activity of AgNPs

Due to the presence of low amounts of G1 and absence of B1 and G2 as evidenced by HPLC, three random groups, group C (toxin + 20 µg AgNPs) as low dose, group F (toxin + 50 µg AgNPs) as moderate dose, and group H (toxin + 70 µg AgNPs) as high dose, were selected for comparative evaluation for the next in vivo experimental phase. Contaminated wheat grains from these flasks were added to the feed of albino rats to examine the in vivo antifungal activity of AgNPs.

#### 3.5.1. Effect on Body Weight, Feed Consumption, and Feed Conversion Ratio in Albino Rats

A significant decrease in weight gain, feed consumption, and feed conversion ratio were observed in rats fed with only aflatoxins (group B) in comparison to the normal/control group (Table 2). Weight gain in group C (toxin + 20 µg AgNPs), group D (toxin + 50 µg AgNPs), and group E (toxin + 70 µg AgNPs) was substantially higher than in group B. A significant decline (*p* < 0.05) in feed consumption was observed in the aflatoxicated group. Group C also showed a better ratio of feed consumption; however, the best results were achieved in the case of group D (toxin + 50 µg AgNPs), with no significant improvement in group E. The ameliorative potential of AgNPs was evident from the FCR ratio. The FCR group of rats treated with 20 µg AgNPs and 50 µg AgNPs was significantly improved than the aflatoxicated group. The findings of the current study revealed that rats administered aflatoxins consumed less feed and gained less weight than rats in other groups. Group E showed no discernible change in FCR, which may be related to the toxic effects of the large dose of AgNPs. Weight gain in group D (toxin + 50 µg AgNPs) was observed to be the highest with impaired FCR in the aflatoxicated group. Saleh et al. [101] also reported a decrease in body weight and FCR upon exposure to aflatoxins in rats. Similar results were presented by Hussain et al. [102], and our results are in accordance with it. Hassan et al. [103] reported an improvement in FCR in ochratoxicated albino rats upon administration of 25 ppb ZnONPs.

#### 3.5.2. Effects on Hematological Parameters

The current investigation showed that exposure to aflatoxins affected the majority of the hematological indices in aflatoxicated rats. Compared to control/group A, RBC, WBC, Hb content, HCT, MCV, MCHC, and platelets were considerably lower in aflatoxicated/group B animals (Table 3). The reduction in RBCs, Hb, HCT, MCV, and MCHC may result from decreased erythropoiesis and hemosynthesis caused by heme biosynthesis enzymes being inhibited in activity or by increased erythrocyte breakdown in hematopoietic organs. The production of epinephrine during aflatoxin-induced stress may be responsible for the drop in WBC count, as this hormone also causes the spleen to constrict and decrease the leucocyte count. A decline in WBC may also suggest decreased resistance in rats administered with aflatoxins. Reduced platelet count in rats exposed to aflatoxins implies bone marrow suppression [104]. Sampathkumar et al. [105] and Singh et al. [106] also reported a decrease in RBCs, WBCs, Hb content, and platelet count upon exposure to aflatoxin B1 in albino rats, and our results are in good agreement with it. Likewise, HCT, MCV, and MCHC levels were found to be depressed by aflatoxins in broilers as per the studies reported by Kececi et al. [107] and Oguz et al. [108]. The levels were however found to be improved in group C (toxin + 20 µg AgNPs) and group D (toxin + 50 µg AgNPs) with a subsequent decline in the concentrations again in the case of group E (toxin + 70 µg AgNPs), thus proving the dose of 70 µg AgNPs to be toxic. Ahangaran and Jahromi [109] also showed the therapeutic potential of nanosilver in overcoming the harmful impacts induced by aflatoxins in chickens.

#### 3.5.3. Effects on Liver Function Parameters

**Figure 7:**
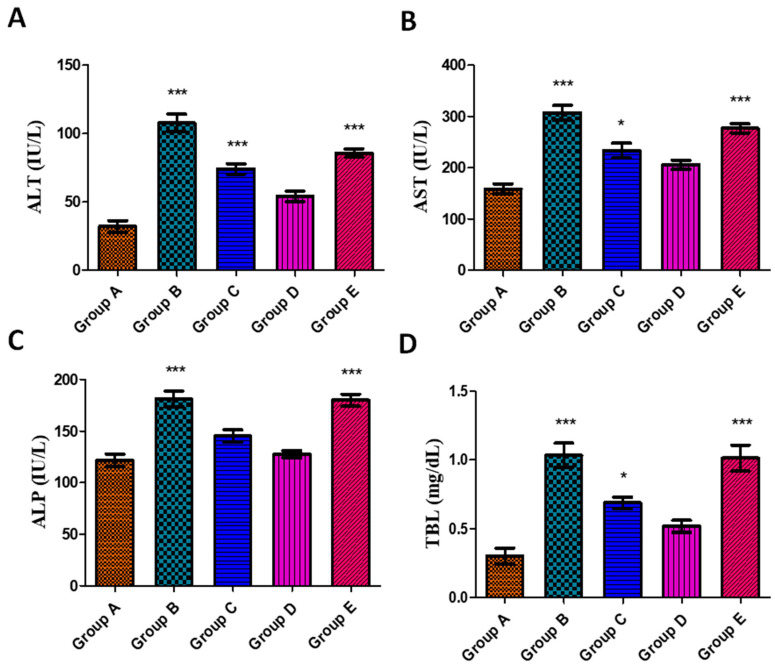
Effect of *J. regia*-mediated AgNPs on ALT (**A**), AST (**B**), ALP (**C**), and TBL (**D**) levels in serum against aflatoxin-induced toxicity in albino rats. Group A represents control group, group B represents aflatoxicated group, group C represents toxin + 20 µg AgNPs, group D represents toxin + 50 µg AgNPs, and group E represents toxin + 70 µg AgNPs. Data are mean ± SEM where *p* < 0.05. * represents the levels of significance between control/group A vs. other groups.

Liver function parameters, i.e., ALT, AST, ALP, and TBL, were performed in order to evaluate the protective effect of the synthesized AgNPs. In toxin group B, the highest levels of ALT (108 ± 6.49), AST (307 ± 14.6), ALP (181 ± 7.54), and TBL (1.03 ± 0.08) were observed. The harmful impact of aflatoxins on liver hepatocytes is the cause of the elevated levels of ALT, AST, ALP, and TBL as represented in Figure 7A–D. Permeability of the cell membranes is altered when liver cells are injured, thus releasing ALT, AST, and ALP into the bloodstream, resulting in greater enzyme activity. The ALT level is regarded as the golden standard for determining liver damage and is found abundantly in the cytoplasm and mitochondria of the cells [110]. Brinda et al. [111] and Al Ghasham et al. [6] also reported elevated levels of ALT, AST, ALP, and TBL in aflatoxin-induced toxicity, and our results are in accordance with it. A significant improvement in levels of ALT, AST, ALP, and TBL was observed in the treated groups C (toxin + 20 µg AgNPs) and D (toxin + 50 µg AgNPs). However, a decline in elevated levels close to normal was observed in group D. In the case of group E, it was discovered that the levels had increased once again, which might be attributed to the toxic effect of a high dose of AgNPs. Quercetin NPs of 100 µM were found to ameliorate the toxic effect of aflatoxin by lowering the levels of ALT, AST, and ALP in albino rats as per the study reported by Eftekhari et al. [112]. Likewise, Ismaiel et al. [113] also showed the therapeutic potential of curcumin NPs at a dose of 100–200 mg/kg b.w. against zearalenone-induced hepatotoxicity and this is in accordance with our findings. Furthermore, Alamri et al. [114] reported the potential hepatoprotective effects of vanillic-acid-loaded AgNPs against hepatotoxicity induced by CCl_4_ in male rats. The study found that the administration of vanillic-acid-loaded AgNPs led to a restoration of normal biochemical parameters and liver tissue architecture, indicating possible benefits for liver function.

#### 3.5.4. Effects on Kidney Function Parameters

**Figure 8:**
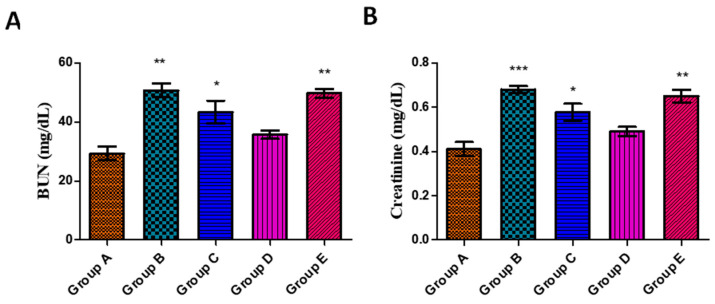
Effect of *J. regia*-mediated AgNPs on BUN (**A**) and creatinine (**B**) levels in serum against aflatoxin-induced toxicity in albino rats. Group A represents control group, group B represents aflatoxicated group, group C represents toxin + 20 µg AgNPs, group D represents toxin + 50 µg AgNPs, and group E represents toxin + 70 µg AgNPs. Data are mean ± SEM where *p* < 0.05. * represents the levels of significance between control/group A vs. other groups.

Kidney function parameters, i.e., BUN and creatinine, were performed in order to evaluate the protective impact of AgNPs against aflatoxin-induced toxicity. Toxin group B showed a significant increase in the levels of BUN (50.7 ± 2.33) and creatinine (0.680 ± 0.015) relative to the control group A as presented in Figure 8A and B, respectively. The higher BUN and creatinine levels are attributed to the damage to the kidney’s proximal tubules upon administration of aflatoxins. However, the concentration of BUN and creatinine was found to be declined in group C (toxin + 20 µg AgNPs) and group D (toxin + 50 µg AgNPs), which can be attributed to the protective effects of *J. regia*-mediated AgNPs. The results showed a raise in levels of BUN and creatinine in group E which may be due to the toxic effect of a higher dose of AgNPs. Similar findings were reported by El-Mahalaway [115] upon the administration of curcumin at a dose of 200 mg/kg b.w. orally for 5 days/week for 4 weeks against experimentally induced aflatoxicosis, and our results are in accordance with it. Likewise, Abdel-Wahhab et al. [116] also showed a decline in the levels of BUN and creatinine upon administration of curcumin NPs loaded hydrogels (100–200 mg/kg b.w.) against aflatoxin-B1-induced toxicity in rats. Biruntha et al. [117] also recently reported the potential antioxidant, hepatoprotective, and nephroprotective effects of AgNPs synthesized from leaf extract of *Tinospora cordifolia*. The findings indicated that the treatment with AgNPs resulted in significant restoration of normal serum levels of urea, creatine, and uric acid in Swiss albino mice that had been induced with potassium bromate. Additionally, the histopathological examination of kidney tissues was consistent with the biochemical results. Overall, the study suggested that the synthesized AgNPs could potentially be used as a therapeutic agent for their strong antioxidant, hepatoprotective, and nephroprotective properties.

#### 3.5.5. Effects on Serum Lipid Profile

**Figure 9:**
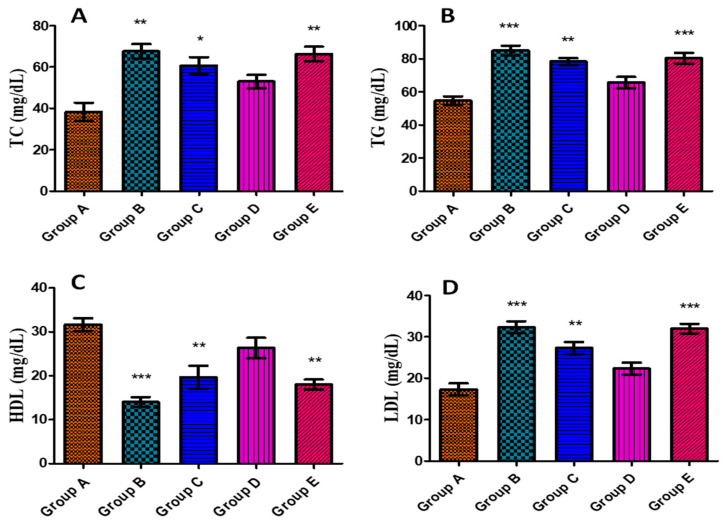
Effect of *J. regia*-mediated AgNPs on serum lipid profile against aflatoxin-induced toxicity in albino rats: (**A**) TC, (**B**) TG, (**C**) HDL, and (**D**) LDL. Group A represents control group, group B represents aflatoxicated group, group C represents toxin + 20 µg AgNPs, group D represents toxin + 50 µg AgNPs, and group E represents toxin + 70 µg AgNPs. Data are mean ± SEM where *p* < 0.05. * represents the levels of significance between control/group A vs. other groups.

Due to disturbed cholesterol metabolism by liver cells, hepatic damage is one of the causes of elevated blood serum cholesterol and triglyceride levels [118]. There have been reports linking increased LDL levels and decreased HDL to hepatic lesions and damage [118,119]. Therefore, to evaluate the liver’s functionality, the lipid profile of the blood serum of experimental animals with aflatoxin-induced toxicity and *J. regia*-mediated AgNPs was examined. According to the findings, blood serum levels of TC, TG, and LDL were significantly increased in groups fed with aflatoxin along with a decline in the levels of HDL (Figure 9A–D). Treatment with toxin + 20 µg AgNPs in group C showed a dip in the levels; however, the differences were not significant. In the case of group D (rats fed with toxin + 50 µg AgNPs), a statistically significant difference was observed in restoring the levels towards normal. Lastly, group E (rats fed with toxin + 70 µg AgNPs) showed a rise in the levels which might be due to the toxic effect of a higher concentration of AgNPs. Our findings show similarity as per the report published by Atef et al. [120] depicting the protective effects of ZnONPs at a concentration from 25 to 50 µg/kg b.w. In addition, El-Nekeety et al. [121] also showed elevated levels of TC and TG in aflatoxin-induced oxidative stress in albino rats.

### 3.6. Histopathology Studies of Organs

#### 3.6.1. Histopathology of Liver

**Figure 10:**
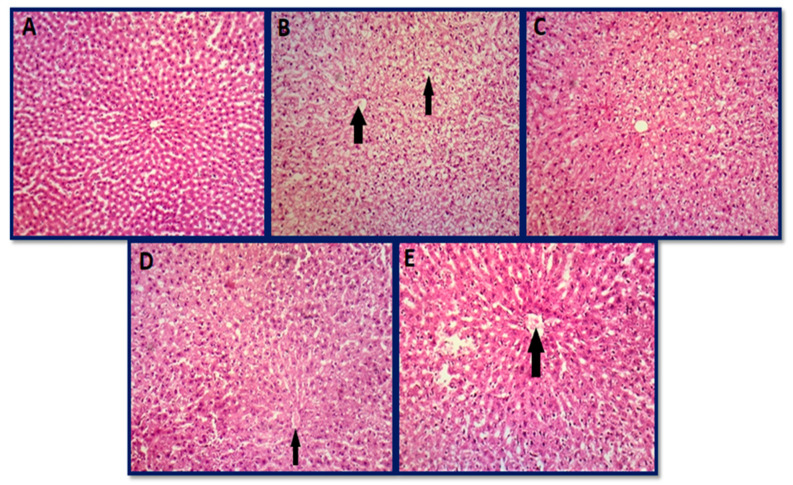
Histopathological photomicrographs of the liver: (**A**) group A (control group), (**B**) group B (aflatoxicated group) showed swelling of hepatocytes with portal infiltration with neutrophils, (**C**) group C (toxin + 20 µg AgNPs) normal architecture, (**D**) group D (toxin + 50 µg AgNPs) showed mild swelling of hepatocytes with distorted hepatic cords as compared to the toxin group, and (**E**) group E (toxin + 70 µg AgNPs) showed mild swelling of hepatocytes and leukocyte infiltration in hepatic cords (hematoxylin and eosin 10×).

The control group A consisting of rats fed with a normal diet demonstrated a normal architecture and decreased sinusoidal spacing (Figure 10A). The major cause of increased liver markers was the necrosis of hepatocytes in the aflatoxicated group B (Figure 10B). Liver architecture improved in treated group C (toxin + 20 µg AgNPs) and group D (toxin + 50 µg AgNPs), as shown by histopathological examination in Figure 10C and D. Group C showed mild swelling of hepatocytes with distorted hepatic cords as compared to the toxin group. The normal architecture was retained with less distortion of hepatic cords in group D as compared to groups B and C. Group E (toxin + 70 µg AgNPs) showed swelling of hepatocytes with distortion of hepatic cords which could be attributed to the toxic dose of AgNPs (Figure 10E). Similar findings were published by Abdel-Raheim et al. [122] demonstrating vacuolar degeneration in cytoplasm along with necrosis and congestion in sinusoids, and our results show good accordance with it. In addition, Banu et al. [123] also reported necrosis, fatty infiltration, and hepatic degeneration in aflatoxin-administered rats, which supports our findings.

#### 3.6.2. Histopathology of Kidney

**Figure 11:**
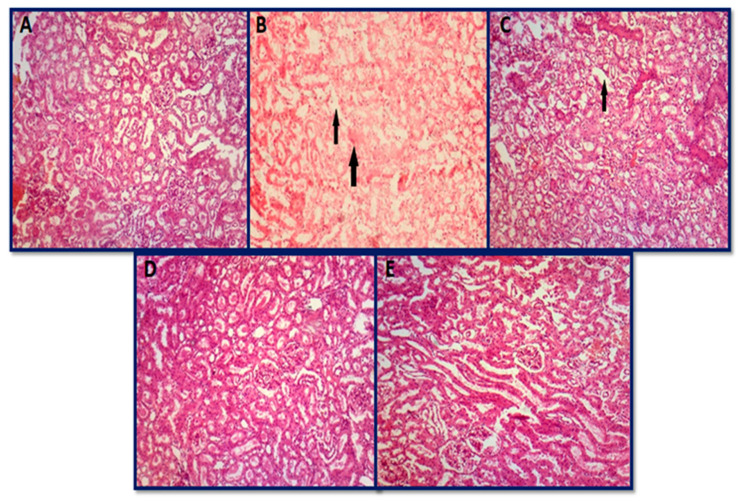
Histopathological photomicrographs of the kidney: (**A**) group A (control group), (**B**) group B (aflatoxicated group) showed necrosis with indistinguishable glomerulus and renal tubules, (**C**) group C (toxin + 20 µg AgNPs) showing mild necrosis, (**D**) group D (toxin + 50 µg AgNPs) normal, and (**E**) group E (toxin + 70 µg AgNPs) normal (hematoxylin and eosin 10×).

The control group consisting of rats fed with a normal diet demonstrated a normal architecture of the kidney with glomeruli surrounded by Bowman’s capsule along with proximal and distal convoluted tubules (Figure 11A). Toxin group B showed coagulated necrosis in tubular epithelial cells as compared to the control (Figure 11B). A significant vacuolation of cytoplasm in tubular epithelial cells was observed in group C (Figure 11C). Normal architecture was retained in group D, thus proving a 50 µg dose of AgNPs as an ideal dose (Figure 11D). Elevated concentration of AgNPs (70 µg) showed toxic effects with mild necrosis and proteinaceous casts in the tubular lumen (Figure 11E). Milicevic et al. [124] also described the vacuolar swelling and degradation in renal tubule epithelial cells. Necrotic alterations in tubular epithelial cells were another finding in the current investigation and were in accordance with the report published by Morsy et al. [125].

#### 3.6.3. Histopathology of Spleen

**Figure 12:**
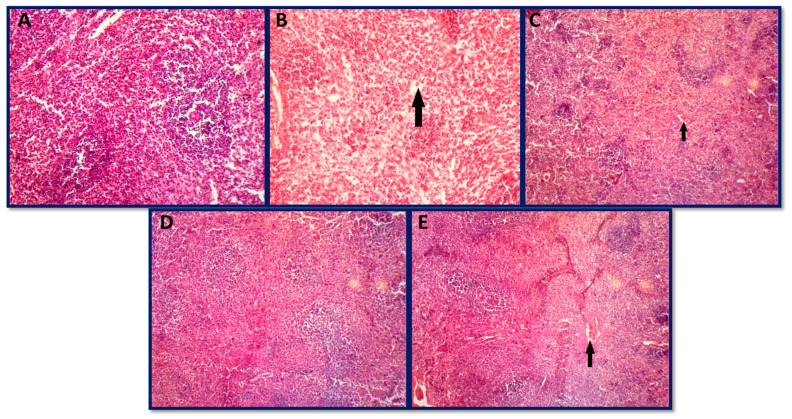
Histopathological photomicrographs of the spleen: (**A**) group A (control group), (**B**) group B (aflatoxicated group) showed necrosis with slightly vacuolated cells, (**C**) group C (toxin +20 µg AgNPs) showed necrosis, (**D**) group D (toxin + 50 µg AgNPs) normal, and (**E**) group E (toxin + 70 µg AgNPs) showed mild necrosis (hematoxylin and eosin 10×).

Group A showed a normal architecture of the spleen with well-defined white pulp and red pulp that surrounds the white pulp (Figure 12A). Toxin group B showed several degraded sites with debris from ruptured degraded cells, and some areas showed necrosis, with slightly vacuolated cells (Figure 12B). Similar findings were reported by [126] and our results are in accordance with it. AFB1 has been shown to alter immunological function in a variety of animal species and is known to suppress the inflammatory response in the liver by inhibiting kupffer cell activation, which is consistent with the decrease in macrophage activity as reported in splenic histopathology previously [127,128]. AgNPs-treated groups (group C and group E) showed altered lymphoid architecture with degraded sites (Figure 12C,E). Group D (toxin + 50 µg AgNPs), however, retained a normal architecture of the spleen as presented in Figure 12D. Kumar and Balachandran [129] also showed necrosis and vacuolation in cells in the spleen upon administration of aflatoxins in broiler chickens, and our results show similarity with it. Peng et al. [130] also reported the damaging effects of aflatoxins on the spleen, which shows good accordance with our findings.

#### 3.6.4. Histopathology of Skeletal Muscle and Heart

**Figure 13:**
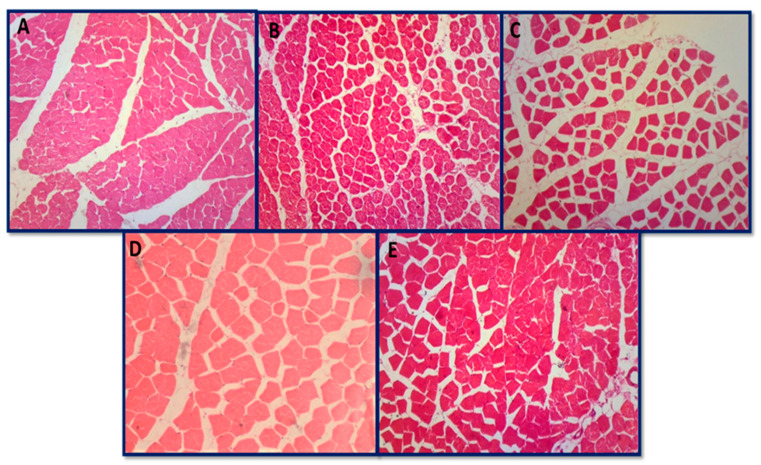
Histopathological photomicrographs of the skeletal muscle: (**A**) group A (control group), (**B**) group B (aflatoxicated group) normal architecture, (**C**) group C (toxin + 20 µg AgNPs) normal architecture, (**D**) group D (toxin + 50 µg AgNPs) normal architecture, and (**E**) group E (toxin + 70 µg AgNPs) normal architecture (hematoxylin and eosin 10×).

**Figure 14:**
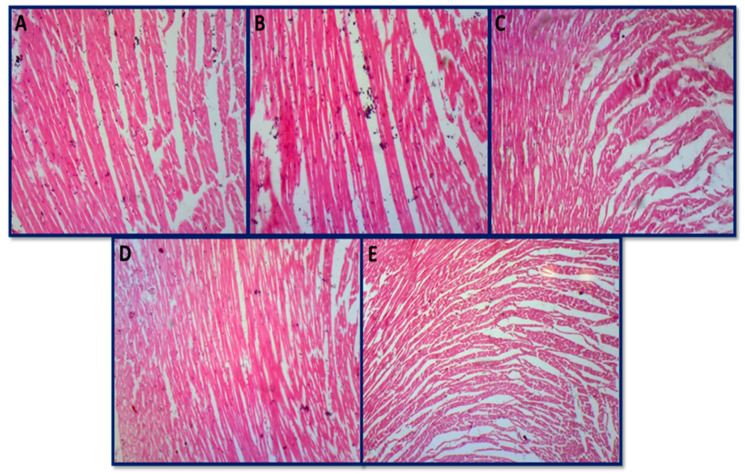
Histopathological photomicrographs of heart: (**A**) group A (control group) normal, (**B**) group B (aflatoxicated group) normal, (**C**) group C (toxin + 20 µg AgNPs) normal, (**D**) group D (toxin + 50 µg AgNPs) normal, and (**E**) group E (toxin + 70 µg AgNPs) normal architecture (hematoxylin and eosin 10×).

No histopathological alterations in skeletal muscles were found in all groups, including control, toxin, and AgNPs-treated groups. All histopathological photomicrographs showed the normal cross-sectional architecture of skeletal muscle fibers along with a polyangular appearance (Figure 13A–E). All histopathological photomicrographs of the heart also showed the normal architecture of cardiac muscle cells with centrally located nuclei (Figure 14A–E). Both the heart and the skeletal muscle showed no significant alterations in the current study across all groups. Biro et al. [131] also reported no evidence of skeletal muscle or cardiac fiber damage caused by aflatoxins, and our results are in accordance with it.

## 4. General Mechanism of Antifungal Activity by AgNPs

The exact mechanism of the antifungal activity of AgNPs is not fully understood, but there are several proposed mechanisms that have been suggested by researchers.

One mechanism is the disruption of the fungal cell membrane. AgNPs can interact with the cell membrane of fungi and cause structural changes, such as the formation of pits, blebs, and invaginations [132,133,134]. This can result in the loss of membrane integrity, leading to leakage of intracellular contents and eventually cell death. Additionally, AgNPs can also penetrate the cell membrane and enter the cell, where they can cause damage to intracellular structures and biomolecules [135,136,137].

Another proposed mechanism is the generation of reactive oxygen species (ROS). AgNPs can produce ROS, which are highly reactive molecules that can cause damage to cellular structures and biomolecules. In fungi, ROS can cause oxidative stress, disrupt cellular homeostasis, and ultimately lead to cell death [138,139].

Furthermore, AgNPs can also interfere with vital cellular processes in fungi. For example, AgNPs can inhibit enzyme activity by binding to active sites of enzymes or by disrupting enzyme–substrate interactions. This can interfere with metabolic pathways and energy production, leading to growth inhibition and cell death [140,141,142].

Overall, the exact mechanism of the antifungal activity of AgNPs is likely to be complex and multifactorial, involving a combination of the above-mentioned mechanisms, as well as other unknown mechanisms.

## 5. Conclusions

From the results, it is concluded that the toxigenic fungus *Aspergillus ochraceus* has severe effects on biochemical and hematological parameters and causes histopathological changes in albino rats. The antifungal activity of AgNPs has been shown to be effective in reducing toxigenic effects in albino rats, as measured by improvements in hematological and biochemical parameters, and histopathological analysis. Since prevention is the most economical and practical course of action, it should be pursued due to the significant role that aflatoxin contamination represents, particularly the detrimental impact of aflatoxins on public health. AgNPs have the potential to be effective options for removing aflatoxin contamination from food and feedstuffs based on their demonstrated abilities to inhibit the growth of mycotoxins-producing *Aspergillus ochraceus*.

In silico/computational simulation studies can also be a valuable tool in detecting aflatoxins in feed and humans. By using advanced computer models and algorithms, researchers can analyze large amounts of data and predict the behavior of aflatoxins in different environments. This can help to identify the most effective and efficient methods for detecting aflatoxins in feed and humans and ultimately improve food safety and public health. Further research is however needed to fully understand the mechanisms underlying the antifungal activity of AgNPs and to optimize their use for practical applications. The safety and environmental impact of AgNPs also needs to be evaluated for reducing aflatoxin contamination in food and feedstuffs. This could involve conducting toxicity studies in animal models and investigating the fate and behavior of AgNPs in the environment to ensure that they do not pose any unintended risks or negative impacts on human health or the environment.

In summary, while the results of this study provide promising evidence of the potential of AgNPs as an effective antifungal agent against *A. ochraceus* and for reducing aflatoxin contamination, further research is needed to fully understand their mechanisms of action, optimize their synthesis and use, and ensure their safety and environmental impact.

## Figures and Tables

**Table 1:** HPLC analysis of aflatoxins (G1, B1, and G2) present in different experimental groups showing the in vitro antifungal activity of AgNPs.

Aflatoxin (ng/mL)	Group A	Group B	Group C	Group D	Group E	Group F	Group G	Group H
G1 (ng/mL)	363 ± 3.06	333 ± 7.57	336 ± 11.5	319 ± 8.00	307 ± 7.64	175 ± 3.06	374 ± 4.04	401 ± 9.02
B1 (ng/mL)	9.74 ± 0.541	8.55 ± 0.415	5.68 ± 0.555	4.71 ± 0.536	1.56 ± 0.555	Absent	Absent	Absent
G2 (ng/mL)	54.4 ± 3.95	35.4 ± 4.44	32.7 ± 2.67	24.4 ± 3.50	Absent	Absent	Absent	Absent

**Table 2:** Effect of aflatoxins and *J. regia*-mediated AgNPs on weight gain, feed consumption, and FCR in albino rats.

Group	Rat Weight (g)	Feed Consumed (g)	FCR
Group A (Control)	650 ± 3.00 ^c^	720 ± 3.00 ^c^	1.10 ± 0.01 ^a,b^
Group B (Toxin)	627 ± 2.00 ^e^	642 ± 3.00 ^e^	1.02 ± 0.01 ^c^
Group C (Toxin + 20 µg AgNPs)	670 ± 2.00 ^b^	730 ± 1.00 ^b^	1.08 ± 0.02 ^b^
Group D (Toxin + 50 µg AgNPs)	692 ± 2.00 ^a^	770 ± 2.00 ^a^	1.11 ± 0.02 ^a^
Group E (Toxin + 70 µg AgNPs)	640 ± 2.00 ^d^	657 ± 2.00 ^d^	1.02 ± 0.01 ^c^

Values were represented as mean ± SD. Various superscripts on the mean represented significant differences (*p* < 0.05) among groups.

**Table 3:** Effects of aflatoxins and *J. regia*-mediated AgNPs on hematological parameters.

**Groups**	**RBCs (×10^12^/L)**	**WBCs** **(×10^9^/L)**	**Hb (g/dL)**	**HCT (%)**	**MCV (fL)**	**MCH (g/dt)**	**Platelets** **(×10^9^/L)**
Group A (Control)	7.9 ± 0.10 ^a^	13.4 ± 0.20 ^a^	13.1 ± 0.20 ^a^	45 ± 2.00 ^a^	61 ± 1.00 ^a^	22 ± 2.00 ^a^	565 ± 1.00 ^a^
Group B (Toxin)	6.3 ± 0.20 ^c,d^	2.5 ± 0.10 ^e^	9.6 ± 0.20 ^e^	34 ± 2.00 ^c^	55 ± 2.00 ^b^	18 ± 1.00 ^b^	200 ± 2.00 ^e^
Group C (Toxin + 20 µg AgNPs)	6.5 ± 0.20 ^c^	10.8 ± 0.10 ^c^	10.9 ± 0.20 ^c^	37 ± 1.00 ^b,c^	56 ± 2.00 ^b^	18 ± 2.00 ^b^	413 ± 1.00 ^c^
Group D (Toxin + 50 µg AgNPs)	7.2 ± 0.10 ^b^	11.2 ± 0.20 ^b^	11.9 ± 0.10 ^b^	40 ± 3.00 ^b^	58 ± 2.00 ^a,b^	20 ± 2.00 ^a,b^	522 ± 2.00 ^b^
Group E (Toxin + 70 µg AgNPs)	6.0 ± 0.20 ^d^	6.7 ± 0.20 ^d^	10.1 ± 0.30 ^d^	35 ± 2.00 ^c^	55 ± 2.00 ^b^	18 ± 1.00 ^b^	400 ± 3.00 ^d^

Values were represented as mean ± SD. Various superscripts on the mean represented significant differences (*p* < 0.05) among groups.

## Data Availability

All data has been provided in this manuscript.
